# High-intensity focused ultrasound ablation combined with systemic methotrexate treatment of intramural ectopic pregnancy: A case report

**DOI:** 10.1097/MD.0000000000031615

**Published:** 2022-11-18

**Authors:** Yan Peng, Yu Dai, Guiyuan Yu, Ping Jin

**Affiliations:** a Department of Gynecology, Affiliated Shenzhen Maternity & Child Healthcare Hospital, Southern Medical University, Shenzhen, Guangdong, China.

**Keywords:** adenomyosis, high-intensity focused ultrasound ablation, intramural ectopic pregnancy, methotrexate

## Abstract

Intramural ectopic pregnancy (IMP) is a rare ectopic pregnancy with an unclear etiology, and standard treatment guidelines currently remain unclear. The main treatment option is local excision of IMP via laparoscopy or laparotomy.

**Patient concerns:**

A 32-year-old woman with adenomyosis presented with amenorrhea for 7 weeks and a serum β-human chorionic gonadotropin (HCG) level of 6882 IU/L. The patient had a history of laparotomy for adenomyosis 5 years previously. Three-dimensional ultrasonography showed a live gestational sac (GS) of 9 × 15 × 18 mm located in the left posterior wall of the uterus and a sinus tract connecting the sac and the endometrial cavity. MRI revealed the GS located in the adenomyosis and a 1.0-cm sinus tract connecting the GS and the endometrial cavity.

**Diagnoses:**

IMP with adenomyosis.

**Interventions:**

High-intensity focused ultrasound (HIFU) treatment combined with systemic methotrexate (MTX) was performed to treat IMP, which would avoid operation and massive bleeding.

**Outcomes:**

Serum β-HCG levels decreased to normal 4 weeks after HIFU treatment and the GS was not found on MRI after 4 months. The sinus tract was significantly shortened after the HIFU treatment.

**Lessons:**

HIFU ablation combined with systemic MTX is effective for the treatment of IMP and is favorable for maintaining fertility.

## 1. Introduction

Intramural ectopic pregnancy (IMP) is characterized by a gestational sac (GS) within the uterine wall that is partially or completely surrounded by the myometrium and is not connected to the fallopian tubes or endometrial cavity.^[1]^ IMP is a rare ectopic pregnancy with an unclear etiology.^[2]^ The majority of IMP cases occur in patients with a history of uterine trauma, such as myomectomy, salpingectomy, dilatation and curettage, assisted reproductive technologies and adenomyosis.^[3]^ Early management of unruptured IMP may provide patients with a chance to preserve their fertility. This report describes a case of IMP in a patient with a history of open adenomyomectomy. To our knowledge, it was the first case treated with high-intensity focused ultrasound (HIFU) ablation combined with the systemic use of methotrexate (MTX). Acceptability was obtained from the Institutional Review Board and Ethics Committee. The patient provided consent for publication of this report.

## 2. Case report

**Figure 1.**
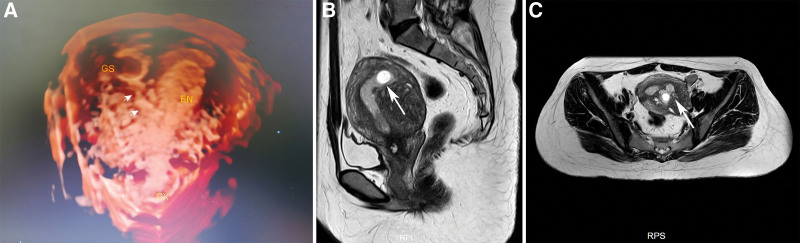
Intramural pregnancy in a 32-year-old woman. (A) Three-dimensional transvaginal showing a GS in the left posterior side of the myometrium and a sinus tract (arrow) connecting the sac and endometrial cavity. (B) Sagittal and (C) transverse views of preoperative T2-weighed images showing adenomyosis and intramural pregnancy sac lesions in the posterior wall of the uterus and a sinus with the length of 1.0 cm (arrows) connecting the sac with the endometrial cavity. GS = gestational sac.

A 32-year-old woman, gravida 1 para 0, was admitted to Shenzhen Maternity and Child Healthcare Hospital on June 12, 2020 for suspected intramural pregnancy. She previously had undergone open adenomyomectomy for adenomyosis in November 2015. The details of the surgical records are unclear. The patient was instructed to have strict contraception for 2 years postoperatively. The patient was treated with 6 cycles of gonadotropin releasing hormone agonists in 2019 because of adenomyomosis relapse. Due to fertility requirement and ovulation disorders, the patient was administered letrozole for ovulation induction after menstruation recovery. The last menstrual period began on April 22, 2020. The serum β-human chorionic gonadotropin (HCG) concentration measured on the 37th day post-amenorrhea was 6463 IU/L. Serum β-HCG level was 6882 IU/L on the 49th day post-amenorrhea and transvaginal ultrasonography scans revealed a gestational sac (GS) measuring 11 × 8 × 13 mm located in the posterior wall of the uterus and an empty endometrial cavity. An echogenic yolk sac and an embryo bud with primordial cardiac pulsation were observed within the GS. Meanwhile, diffuse adenomyosis can be seen on the posterior wall of the uterus. The patient was diagnosed with suspected IMP with adenomyosis by transvaginal ultrasound. Three-dimensional ultrasonography scans identified a live GS measuring 9 × 15 × 18 mm located in the left posterior wall of the uterus and a sinus tract connecting the sac and the endometrial cavity (Fig. 1A). MRI revealed adenomyosis appeared in the posterior wall of the uterus, and a GS also located in the left posterior wall of the uterus and an empty endometrial cavity. The endometrium in the posterior wall of the uterine fundus extended into the myometrium and form a 1.0-cm sinus tract connecting the GS and endometrial cavity. The patient presented with no obvious symptoms. The cyst did not bulge out from the left posterior uterine wall with the shortest distance from uterine serosa of 7 mm (Fig. 1B and C). Thus, the diagnosis of IMP with adenomyosis was confirmed.

Routinely, laparotomy was performed to open the uterus, followed by lesion removal and uterine suturing. As the current case was associated with adenomyosis and the patient had a history of open adenomyomectomy, repeated traditional laparotomy surgery might have caused high trauma and that was still at risk of poor healing in the uterus. The patient strongly resisted another open surgery because of the huge trauma and the long contraceptive time. The patient might have performed hysteroscopy to remove the lesion through the sinus tract. This surgery is required to cut the endometrium and the myometrium around the sinus with a ring electrode to expose the villi,^[4]^ which is not conducive to future pregnancy. Systemic drug therapy with methotrexate was administered. However, as this case involved a live GS, the success rate of MTX treatment alone may be low. HIFU ablation, which was initially developed for the treatment of solid tumors, has been successfully implemented for the treatment of adenomysosis.^[5]^ Many cases of ectopic pregnancy at different sites, such as Caesarean scar pregnancy and cornual pregnancy, have been managed using HIFU ablation, which is a noninvasive therapy.^[6,7]^ No IMP cases have been treated using this approach. Therefore, after a full explanation and communication with the patient, HIFU ablation combined with MTX was selected as the treatment option for her.

**Figure 2.**
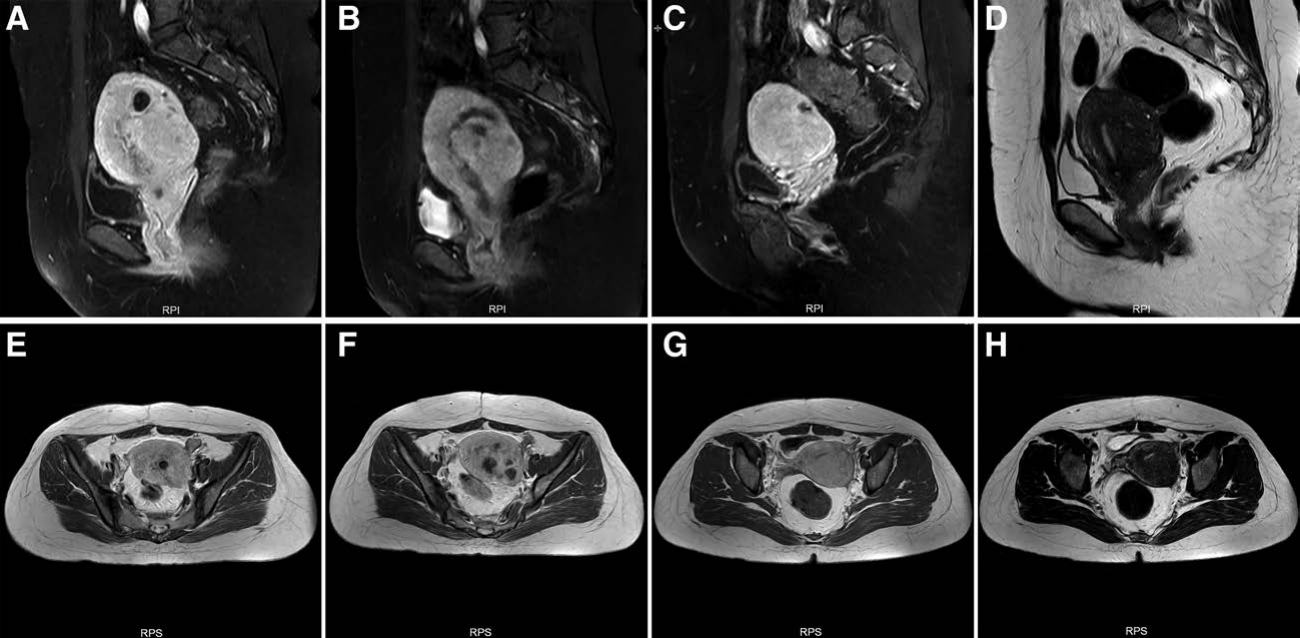
MR image obtained from the adenomyosis patient with IMP. (A) Sagittal and (B) transverse views of preoperative T1-weighed contrast enhanced images showing adenomyosis and intramural pregnancy sac lesions in the posterior wall of the uterus. (C/E) Sagittal and (D/F) transverse views of postoperative T1-weighed contrast enhanced images showing the nonperfused region at the posterior wall of the uterus at post-HIFU (C/D) and 4 months after HIFU (E/F). (G) Sagittal and (H) transverse views of postoperative T2-weighed images only showing adenomyosis lesions in the posterior wall of the uterus 4 months after HIFU. HIFU = high-intensity focused ultrasound, IMP = intramural ectopic pregnancy.

Ultrasound-guided HIFU was performed using the Haifu JC-200D Focused Ultrasound Tumor Therapeutic System (Chongqing Haifu Tech Co., Ltd., Chongqing, China). The patient was required to complete all preoperative preparations before HIFU treatment, including specific skin preparation, bowel preparation, as well as preoperatively administration of fentanyl and midazolam for sedation and analgesia until HIFU treatment ended. The treatment was required to select the sagittal view of the ultrasound scanning model, conduct real-time monitoring on the treatment response and adjust the treatment parameters accordingly. The IMP at the left posterior side of the uterus was treated first, followed by adenomyosis at the posterior side of the uterus. The focus was placed close to the embedding area of the GS in the center slice at the beginning of the HIFU ablation. Then the focus was placed in adenomyosis lesions, and the focal point was at least 1.5 cm away from the margin of uterus and endometrial cavity to prevent intestinal and uterine endometrium damage. The lesion was divided into sections with 3 mm separation. Then, the HIFU treatment power was 400W and the ablation proceeded from the innermost section to the outside section until the entire lesion was covered. The end-point of HIFU ablation was that the blood flow in GS embedding area and adenomyosis area was significantly reduced or grayscale changes was observed on color Doppler ultrasound. The therapeutic effect was confirmed by contrast-enhanced ultrasound with SonoVue (SonoVue, Bracco, Italy) and MRI post HIFU. Total ablation was performed for 578 seconds without bleeding. At the end of treatment, there was a significant decrease in blood supply around the GS in contrast-enhanced ultrasound. On MRI, a slight collapse of the GS was observed, the area around the GS of the IMP showed an estimated non-perfusion volume ratio of 80% approximately, and the effect of HIFU ablation for GS was satisfactory (Fig. 2A–D). The non-perfusion volume ratio of adenomyosis was approximately 15%, and the adenomyosis lesions were partially ablated (Fig. 2A–D). Following the 1st day of HIFU treatment, the patient’s serum β-HCG level had significantly decreased to 2522 IU/L and a 5-day regimen of intramuscular MTX (20 mg/day) injections was prescribed to kill the activity of possibly residual trophoblast cells. Serum HCG levels decreased to 1792 IU/L and 857 IU/L on day 1 and day 4, respectively, after MTX treatment. No complications were found during HFU or MTX treatment. The patient was then discharged from the hospital.

**Figure 3.**
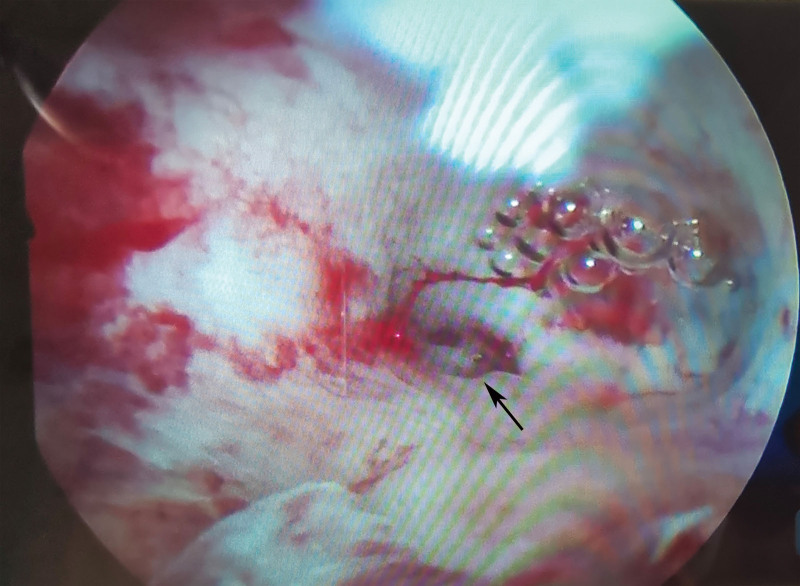
Hysteroscopy revealing a uterine sinus tract (3 × 3 × 3 mm) (arrow)in the left posterior wall of uterus near the corner 4 months later after HIFU. HIFU = high-intensity focused ultrasound.

The serum β-HCG level returned to normal after 4 weeks. Dysmenorrhea of the patient was partially relieved after HIFU treatment. MRI did not identify the sinus tract and GS in the posterior wall of the uterus and only revealed adenomyosis in the posterior wall of the uterus (Fig. 2E–H) 4 months after HIFU treatment. Meanwhile, a hysteroscopic examination was conducted, revealing that the left posterior wall of uterine fundus near the corner had a uterine sinus tract (3 × 3 × 3 mm) with its base communicating to the uterine cavity. The “sinus tract” was not found on MRI yet proved by hysteroscopy (Fig. 3). The length of sinus tract after HIFU treatment is shorter than that before HIFU treatment. Adenomyosis was still diffuse in the posterior wall of the uterus. The uterine sinus tract was considered to be a scar defect after adenomyomectomy. Owing to the small size of sinus tract and its location in adenomyosis combined with the patient’s personal wishes, we did not perform repeated surgery to remove the sinus tract. She began to try pregnancy 4 months after HIFU treatment. Ten months after HIFU treatment, the patient was pregnant again by monitoring the follicle and the GS was located in the uterine cavity 51 days after menopause. An echogenic yolk sac and an embryo bud with primordial cardiac pulsation were observed within the GS. The shortest distance between the GS and the uterine serous membrane was 2 cm. One week later, transvaginal ultrasound revealed the GS without primordial cardiac pulsation and diagnosed missed abortion.

## 3. Discussion

The pathogenesis of IMP remains unclear. The possible risk factors include prior uterine trauma, adenomyosis, pelvic surgery, and in vitro fertilization.^[1]^ In our case, the patient had a history of adenomyomectomy 5 years previously. Sonography and MRI clearly showed that the sinus tract was connected to the sac and endometrial cavity, and hysteroscopy also confirmed the presence of sinuses, which proved the sinus tract in our case was formed traumatically during a prior adenomyomectomy and further supported the viewpoint that IMP can result from previous uterine trauma. This case leaves many issues associated with treatment among patients of reproductive age with adenomyosis open for discussion. There is no clear and unified standard for how to treat patients with adenomyosis who have fertility requirements.^[8]^ The patient in our case without infertility and miscarriage underwent surgical management of adenomyosis in 5-year prior, which increased her risk of future complications including ectopic pregnancy, placental adhesive disorders and uterine dehiscence in future pregnancies. Patients with adenomyosis who have fertility requirements should carefully choose surgical treatment.

Because of the rarity of IMP, standard treatment guidelines currently remain unclear. However, once the diagnosis is established, appropriate treatment should be carried out without delay before complications occur. The management options include medical therapy and surgery. In the past, hysterectomy or surgical resection was used to manage uterine rupture and severe hemorrhage secondary to IMP. With the early diagnosis of IMP, there are a variety of conservative management options for IMP, including local or systemic injection of methotrexate and laparoscopic or hysteroscopic lesion resection.^[4,9,10]^ It has been suggested that the management of IMP should be individualized based on the lesion location, extent of myometrial involvement, gestational age at diagnosis, the patient’s condition and wishes for future fertility, the clinician’s judgment, as well as facilities available.^[9]^ Medical therapy is rarely used alone and often combined with conservative surgical treatment. The main surgical option is local excision of the IMP via laparoscopy or laparotomy and these main concerns are the long period of contraception and the subsequent risk of uterine rupture. Hysteroscopic surgery might be a choice if imaging techniques identify a sinus linking the GS with the uterine cavity,^[4]^ which will damage the structure of the uterus and is not conducive to subsequent pregnancy.

HIFU ablation is a noninvasive therapy, which could make the in vitro low-energy ultrasound to be focused selectively on target tissues in the body, lead to a heat over 65℃, then induce coagulative necrosis, and spare the adjacent normal tissues at the same time. HIFU also can damage small vessels with a diameter less than 2 mm,^[11]^ which leads to reduce the blood supply around the GS in our patient. Several studies have examined the safety profile of ultrasound-guided HIFU in treating patients with adenomyosis, besides sustaining long-term clinical improvements and achieving better postoperative reproductive outcomes.^[12]^ HIFU ablation has also been performed to treat cesarean scar pregnancies and cornual ectopic pregnancy.^[6,7]^ It is confirmed that HIFU could terminate cesarean scar pregnancies with no obvious adverse effects on subsequent pregnancy. These studies indicates the feasibility of HIFU for the treatment of ectopic pregnancy lesions in uterus, though no case in which a patient with IMP was treated by HIFU treatment has been reported previously. Here we report that IMP with a history of open adenomyomectomy could be successfully managed by HIFU combined with MTX during early pregnancy, which is less traumatic and capable of preserving the integrity of the uterus and has a short contraceptive time.

The particularity of this case is the concurrent adenomyosis, which was located at the posterior wall of the uterus and the IMP lesion was wrapped within the adenomyosis. The sinus tract linked the GS with the uterine cavity, which was resulted from previous adenomyomectomy. Local excision of the IMP via laparoscopy or laparotomy can simultaneously remove the sinus tract, but the contraceptive time is long. The diffuse adenomyosis lesions may also lead to poor healing of uterine incision and sinus formation again. HIFU treatment not only ablated ectopic pregnancy lesions, but also ablated a small part of adenomyosis lesions at the same time. However, the treatment of HIFU combined with MTX couldn’t remove the sinus tract. In the end there is an unexpected discovery that the sinus tract was significantly shortened after HIFU treatment. The possible reason is that the ectopic endometrial tissue in the sinus tract was also ablated by HIFU, which may lead to remodel of the sinus structure and shortening of the sinus tract. In our case, the patient’s second pregnancy located in the uterine cavity. HIFU treatment might be helpful for subsequent pregnancy because of the shortening of the sinus tract. Unfortunately missed abortion occurred in the end. Pregnancy patients with adenomyosis have a high rate of miscarriage.^[13]^ Therefore, pregnancy related to adenomyosis is a very difficult problem.

## 4. Conclusion

HIFU, a novel noninvasive therapeutic method, combined with MTX treatment is effective for the treatment of IMP and favorable for maintaining fertility. Hopefully, our findings will play an enlightening role and attract the attention of clinicians.

## Author contributions

**Data curation:** Yan Peng.

**Formal analysis:** Yan Peng, Yu Dai.

**Investigation:** Yan Peng, Guiyuan Yu.

**Methodology:** Yan Peng, Yu Dai, Guiyuan Yu.

**Project administration:** Yan Peng, Ping Jin.

**Resources:** Yan Peng, Yu Dai.

**Supervision:** Ping Jin.

**Writing – original draft:** Yan Peng.

**Writing – review & editing:** Yan Peng.
